# Anti-Angiogenic Properties of Ginsenoside Rg3

**DOI:** 10.3390/molecules25214905

**Published:** 2020-10-23

**Authors:** Maryam Nakhjavani, Eric Smith, Amanda R. Townsend, Timothy J. Price, Jennifer E. Hardingham

**Affiliations:** 1 Molecular Oncology, Basil Hetzel Institute for Translational Health Research, The Queen Elizabeth Hospital, Woodville South, SA 5011, Australia; maryam.nakhjavani@adelaide.edu.au (M.N.); eric.smith@adelaide.edu.au (E.S.); 2 Adelaide Medical School, University of Adelaide, Adelaide, SA 5005, Australia; amanda.townsend@sa.gov.au (A.R.T.); timothy.price@sa.gov.au (T.J.P.); 3 Medical Oncology Unit, The Queen Elizabeth Hospital, Woodville South, SA 5011, Australia

**Keywords:** ginsenoside Rg3, 20(S)-ginsenoside Rg3, 20(R)-ginsenoside Rg3, angiogenesis, epimer

## Abstract

Ginsenoside Rg3 (Rg3) is a member of the ginsenoside family of chemicals extracted from *Panax ginseng*. Like other ginsenosides, Rg3 has two epimers: 20(S)-ginsenoside Rg3 (SRg3) and 20(R)-ginsenoside Rg3 (RRg3). Rg3 is an intriguing molecule due to its anti-cancer properties. One facet of the anti-cancer properties of Rg3 is the anti-angiogenic action. This review describes the controversies on the effects and effective dose range of Rg3, summarizes the evidence on the efficacy of Rg3 on angiogenesis, and raises the possibility that Rg3 is a prodrug.

## 1. Introduction

**Figure 1:**
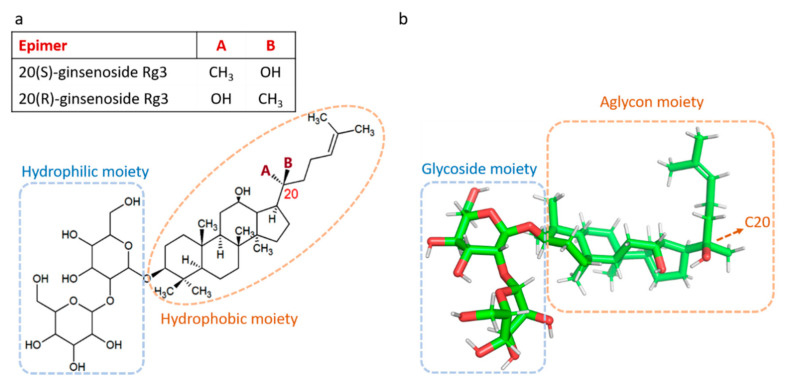
Structure of ginsenoside Rg3 as 2D (**a**) and 3D, generated in UCSF Chimera program (**b**), showing the chiral center at carbon 20, aglycone steroid-like backbone with hydrophobic properties and glycoside hydrophilic moiety, responsible for the water solubility of ginsenoside Rg3 (Rg3).

The root of the plant *Panax ginseng* C.A. Meyer, commonly known as ginseng, has been used as a traditional medicine in Asian countries for thousands of years. It was primarily used as a food and source of energy and strength. Gradually several pharmacological effects of ginseng on immune function, cardiovascular system, neurological disorders and cancer treatment were discovered [1]. The major bioactive components of ginseng responsible for its pharmacological action are ginsenoside saponins. The general structure of ginsenosides is a four-ring steroid backbone with hydrophobic properties, which is connected to sugar molecules, responsible for the hydrophilicity of the molecule. Based on the positioning of hydrogen on carbon 20 (C20), ginsenosides have two stereoisomers; 20(S) and 20(R) epimers. Ginsenoside Rg3 (Rg3) is a member of the ginsenoside family of saponins, and like other members, Rg3 has two epimers, 20(S)-ginsenoside Rg3 (SRg3) and 20(R)-ginsenoside Rg3 (RRg3) (Figure 1).

Steam heating the white fresh ginseng for several hours prepares red ginseng which has improved pharmacological efficacy and is enriched for some ginsenosides including Rg3 [2,3]. This process produces mainly SRg3 as the major epimer. Furthermore, enzymatic hydrolysis [4,5] or alkali hydrolysis [6] are other methods of preparation of SRg3. However, production of RRg3 requires procedures that are more complex [7]. The quantity of Rg3 in red ginseng is very much dependent on the method of preparation and various methods have resulted in various contents, for example, 1.2 mg/mL Rg3 was recovered by *Phellinus linteus* fermentation method [8]. The steaming condition also results in different amounts of Rg3 as reported by different studies such as 25 µg/mL [9], 39 mg/g [2] or 0.28% *w*/*w* [10].

Rg3 is one of the most studied and pharmacologically active ginsenosides, with stereoselective activities by the epimers SRg3 and RRg3 [11,12]. The chemistry of Rg3 epimers could explain this stereoselective activity. For example, stereoselective activity of Rg3 epimers in interaction with Na^+^ channels has been described [13]. Positioning of hydroxyl on C20 seems to play an important role in the pharmacological effects of Rg3. The alkene chain in the aglycone moiety of Rg3 (Figure 1) produces a tight hydrophobic packing near C20 which makes it inaccessible to water molecules, facilitates hydrophobic bonding between SRg3 and Na^+^ ion channels and makes a more stabilized hydrogen binding between the two [13].

One of the important properties of Rg3 is its anti-cancer properties. The mechanisms of Rg3 in inhibition of proliferation, migration and invasion of cancer cells was reviewed previously [11]. Angiogenesis plays a major role in the growth and metastasis of a tumor and one of the important properties of Rg3 is its action on angiogenesis. This review paper aims to look at the different aspects of anti-angiogenic properties of Rg3, using PubMed as the search engine with Mesh terms ginsenoside Rg3 and angiogenesis for all published papers between 1995 and 2020. The first study demonstrating the anti-angiogenic properties of Rg3 was published by Mochizuki et al. in 1995 [14]. They showed in a mouse model of metastatic melanoma that 100 µg/mouse intravenous (i.v.) or 300 µg/mouse oral (p.o.) of either epimer inhibited the formation of vessels oriented towards the tumor mass. This animal study was, however, a short-term study (6 days), with only three mice per group. It was a remarkable study in the area since it not only demonstrated the anti-angiogenic potential of Rg3 in vivo, but also tested both epimers, separately [14]. This is especially important since most of the research published on Rg3 has not described which specific epimer was studied. Since then, several studies have been conducted in vitro and in vivo, which are reviewed here.

## 2. The Controversies on the Effects of Rg3 on Angiogenesis

Studying the proliferation and tube formation of human umbilical vein endothelial cells (HUVECs) on a layer of Matrigel is the mainstay of drug studies investigating anti-angiogenic properties. With Rg3, both of these aspects are a matter of controversy. A few studies have shown that the effective concentration of Rg3 for inhibition of loop formation was at nM ranges (Table 1) [15,16,17,18,19]. For example, RRg3 at 1–1000 nM inhibited tube-formation and chemotactic migration of HUVECs. At this concentration, RRg3 also decreased microvascular sprouting and hemoglobin content of tumors (in a Matrigel plug assay) [15]. Concentrations as low as 1.3 µM Rg3 (not as a specific epimer) inhibited tube-forming capacity of HUVECs and hemoglobin content of Matrigel plugs [16]. At 60 and 300 ng/mL, Rg3 showed effectiveness in inhibition of differentiation of endothelial progenitor cells (EPCs) [18], though it did not inhibit the proliferation of these cells [17]. Although these studies showed the effectiveness of nM concentrations of Rg3, other studies tested higher doses at µM scale and in most cases showed anti-angiogenic properties. The exceptions are the studies that suggested Rg3 at µM concentrations was proangiogenic (Table 1) [20,21].

The study by Kwok et al. showed that 15 µM of SRg3 and RRg3 increased the rate of proliferation by 50 and 10%, respectively. Only SRg3 induced DNA synthesis (15 µM) and migration of HUVECs (15–30 µM). SRg3 and to a lower degree RRg3, increased loop formation in HUVECs. Exposing the cells with SRg3 and not RRg3 led to a prompt and continuous activation of extracellular signal-regulated kinase (ERK) followed by activation of Akt (phosphorylation at Ser473) and endothelial nitric oxide synthase (eNOS) (phosphorylation at Ser1177). It also showed that these two epimers, stereoselectively and with different potencies, interact with and activate peroxisome proliferator-activated receptor-gamma (PPARγ) [20]. PPARγ is one of the ligand-dependent transcriptional factors with polyunsaturated fatty acids as its endogenous ligands. One of the roles of PPARγ is in regulating angiogenesis [20,24] and they showed that the activation of ERK/Akt/eNOS pathway by Rg3 is dependent on the activation of PPARγ. It is noteworthy that in this study, instead of vascular endothelial growth factor (VEGF) as a supplement for the growth of HUVECs, fetal bovine serum was used. This might explain the observed controversy in the literature (see Section 3).

Other studies showed anti-angiogenic effects of Rg3 at µM range. At 65 µM, Rg3 inhibited tube formation and migration. This inhibition was associated with decreased protein and transcript expression of vascular endothelial growth factor (VEGF), basic fibroblast growth factor (b-FGF) and matrix metalloproteinase-2 (MMP-2) and protein expression of MMP-9 [22]. The anti-angiogenic properties of Rg3 were also studied in combination with temozolomide. Temozolomide is one of the effective drugs to improve survival rate and progression-free survival of glioblastoma patients. In a study by Sun et al., the in vitro data suggested that the combination of the oral chemotherapeutic temozolomide (10 µg/mL) and Rg3 (10 µg/mL) had additive effects on inhibition of HUVEC proliferation [23]. At 180 µg/mL, temozolomide and 180 µg/mL Rg3 (144 h), inhibition of proliferation was observed in HUVECs. This combination also decreased the transcript expression of VEGF and Bcl-2, a regulator of apoptosis that inhibits the function of proapoptotic proteins, in HUVECs [23].

Other than the reported controversy about the pro- or anti-angiogenic effect of Rg3 at µM range, some studies have not shown an anti-proliferative effect of Rg3 on HUVECs. For example, 50 µg/mL Rg3 did not inhibit the proliferation of HUVECs within 72 h [22] and the anti-proliferative effect at 1–1000 nM, while significant, was very weak and not dose-dependent [15]. A time- and dose-dependent inhibition of proliferation of HUVECs was reported with Rg3 (0–180 µg/mL). At 180 µg/mL (144 h) about 28% inhibition of proliferation was observed. Rg3 at these concentrations induced S-phase cell cycle arrest (not time-dependent). Exposure of HUVECs for 72 h to Rg3 (80 µg/mL) decreased the expression of VEGF and Bcl-2 [23].

## 3. Pharmacodynamic Aspects of the Effect of Rg3 on Angiogenesis

To address the question about controversies on the effects of Rg3 on angiogenesis at various concertation ranges, the possible explanations might depend on the pharmacodynamics of the interaction of Rg3 with its receptors. VEGF is the main ligand to its receptor, VEGFR2, the interaction of which plays the key role in angiogenesis. Any full agonist binds to the same binding site of VEGF on VEGFR2 and mimics the action of VEGF, leading to a maximal effect (Emax).

One possible explanation could be that Rg3 might be a partial agonist at nM concentrations. A partial agonist, in the absence of an agonist activates the receptor, while in the presence of agonist acts like an antagonist. In vitro assays with endothelial cells usually use a constant concentration of VEGF in the media. This concentration is usually low and at the levels of ng/mL. At nM concentrations, Rg3, if considered as a partial agonist, and in the presence of a constant level of VEGF, might have a role of a competitive antagonist for VEGFR2. At higher concentrations it could act as an agonist of the receptor. Two examples of the anti- and pro-angiogenic effects of Rg3 on HUVECs were discussed above. At nM concentrations and in the presence of VEGF, RRg3 showed anti-angiogenic affects [15] and at low µM range (up to 30 µM) in the presence of fetal bovine serum, Rg3 had pro-angiogenic effects [20]. Therefore, at nM range and in the presence of VEGF, Rg3 acted as an antagonist and in the absence of VEGF acted as agonist.

The other explanation is the possibility of Rg3 having a biphasic U-shaped dose-response curve. In that case, Rg3 would be one of the many examples of molecules having such a biphasic dose-response curve. Examples of such molecules are estrogens [25], NO [26], cadmium and mercury [27], opiates [28], dopamine [29], and anti-angiogenic agents such as endostatin [30], statins [31], captopril [32] and interferon-alpha [33].

However, as described above and previously reviewed [11], there are many studies that used and showed the efficacy of Rg3 at high µM ranges up to 230 µM (180 µg/mL) [23]. This opens another window for Rg3 to have a triphasic dose-response. Examples of molecules with triphasic dose-response are vasopressin [34], neurotensin [35] and amphetamine [36].

## 4. Molecular Mechanisms of Rg3 in Targeting Angiogenesis

When the balance between pro- and anti-angiogenic agents shifts towards pro-angiogenic agents including VEGF, as a fundamental player, and other factors such as b-FGF, epidermal growth factor (EGF), transforming growth factor β (TGF-β), tumor necrosis factor-alpha (TNF-α), angiogenin, angiopoietin, and interleukin 8 (IL-8) [37], several intracellular pathways are triggered leading to activation of endothelial cell proliferation and migration towards the tumor. Migration of endothelial cells is a complex process which requires coordination of several cellular components and changes the dynamic of cellular compartments. Below, the molecules and signaling pathways that are affected following administration of Rg3 are discussed.

### 4.1. VEGF and its Receptor, VEGFR2

**Figure 2:**
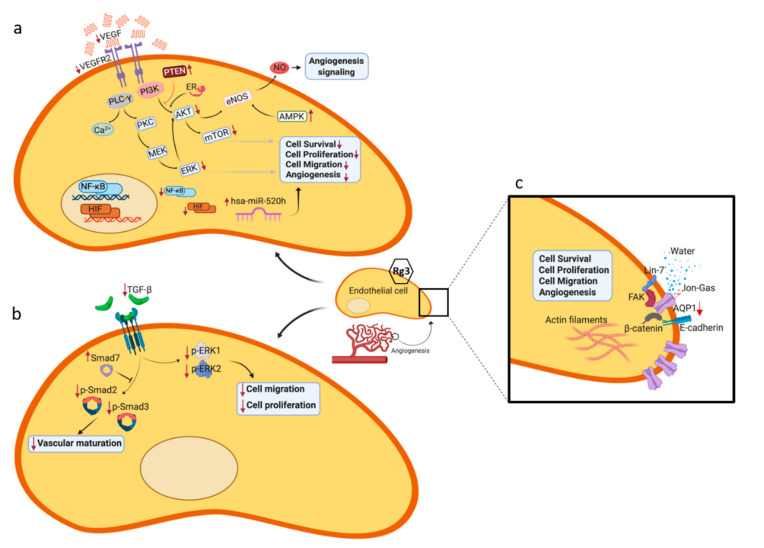
Signaling molecules and pathways that are affected by Rg3 in an endothelial cell. (**a**) VEGF–VEGFR2 interaction and inhibition of the related signaling pathways and molecules, (**b**) decreased expression of TGF-β1 and the related signaling molecules, (**c**) blocking the water transport function of AQP1 and decreased expression of AQP1. Red arrows ↓ and ↑ show the effect of Rg3 on decreased and increased expression of molecules, respectively.

VEGFR2, a receptor tyrosine kinase (RTK), is one of the three subtypes of VEGF receptor. The interaction between VEGF and VEGFR2 is known as the key driver of angiogenesis (Figure 2a). One of the commonly described mechanisms of inhibition of angiogenesis is decreased expression or availability of VEGF and VEGFR2. Rg3 inhibited the protein expression of VEGF in human hepatocellular (HepG2) [38], esophageal (Eca-109) and renal cell carcinoma (786-0) cell lines [39], decreased VEGF-A and -C in anaplastic thyroid and papillary thyroid cancer cell lines [40] and decreased transcripts of VEGF-A, -B and -C in a mouse model of breast cancer [25]. In hypertrophic scar fibroblasts, RRg3 inhibited the transcript and protein expression of VEGF [41]. Likewise, a decreased expression of VEGFR2 was shown in EPCs [17]. Many in vivo studies also showed a decreased expression of VEGF and VEGFR2 (Table 2). The mechanisms involved in such decreased expression of these factors could be explained by the inhibitory action of Rg3 on the expression of hypoxia inducible factor-1α (HIF-1α), cyclooxygenase-2 (COX-2) and nuclear factor-κB (NF-κB) [39]. The VEGF promoter has a hypoxia-responsive element which upon binding to HIF-1α, activates the expression of VEGF [42]. Hypoxia also regulates the expression of COX-2, the expression of which correlates with VEGF [43]. NF-κB is a regulator of various cellular processes that lead to tumorigenesis and metastasis. Angiogenesis is one of these processes. P65 is one of the important members of NF-κB family, the expression of which was inhibited by Rg3 [39].

At least four major downstream intracellular signaling pathways are involved in VEGFR2 activation (reviewed in [44]). The major pathway is the activation of phospholipase Cγ, which can activate a number of downstream signaling molecules and pathways including protein kinase C/Raf/MEK/ERK [45,46]. Activation of this pathway leads to cell proliferation, survival and migration. Another pathway is PI3K/Akt/mTOR pathway which is involved in cell survival and regulation of migration [47]. The third signaling pathway includes SRC and small GTPases that are involved in cell polarization, shape and migration [48]. A fourth signaling pathway involves molecules downstream of VEGFR2 activation: stress kinases such as STATs, G protein-coupled receptor-dependent signaling and p38 MAPK [44]. The specific action of Rg3 on some of the pathways has been elucidated (Figure 2); some of the explored signaling pathways which play roles in angiogenesis are described below.

### 4.2. Signaling Pathways Leading to Activation of eNOS

eNOS is one of the important mediators of angiogenesis (Figure 2a) [62]. It was shown that VEGF-induced activation of phosphatidylinositol 3-kinase (PI3K) activates eNOS by phosphorylation at Ser1177 [63]. Akt is one of the major kinases downstream of PI3K, which is activated following VEGF stimulation and plays a role in cell survival [64]. Activated Akt also directly signals activation of eNOS (Figure 2a) [65]. It was shown that Rg3 (300 ng/mL) decreased VEGF-dependent Akt/eNOS signaling in EPCs [18]. The effect of ginsenosides [66] and Rg3 [67] on NO production was shown previously. Controversies on the effect of Rg3 on eNOS and NO production exist: at 10 µg/mL, increased NO production was reported to be independent of eNOS in canine carporal smooth muscle [67], however in human ECV 304 endothelial cells, the same concentration of Rg3 increased expression and phosphorylation of eNOS via estrogen receptor (ER)-mediated activation of phosphatidylinositol 3-kinase (PI3-kinase) [21]. Involvement of eNOS for production of NO in the Rg3-treated cells might be a tissue- and species-dependent factor. What is controversial here is whether Rg3 increases or decreases the activation of eNOS in endothelial cells. It seems that at 300 ng/mL, the activity of eNOS was decreased [18] while at 10 µg/mL, this activity was increased [21]. Once again it seems that the effect of Rg3 is dependent on the range of concentration. At nM ranges, the activity of eNOS was decreased and at µM ranges, the activity increased. Another regulator of this pathway is a tumor suppressor, phosphatase and tensin homolog deleted on chromosome 10 (PTEN). PTEN is an inhibitor of the PI3K/Akt pathway. In a mouse model of hepatocarcinoma, the mice receiving 5 mg/kg SRg3 showed a non-significant increase in PTEN and decrease in pAkt, as evidenced by immunohistochemistry staining of the tumors. These changes were potentiated and statistically significant when SRg3 was co-administered with sorafenib [68].

The other pathway for the activation of eNOS is via the ER-mediated activation of PI3K/Akt in endothelial cells (Figure 2a) [69] and Rg3 at 10 µg/mL activates this pathway [21]. It is not yet examined whether Rg3 has a similar pattern of response at other ranges of concentration. It is noteworthy that the promoter region of VEGF gene has an estrogen response element (ERE) [70] and the expression of VEGF is affected by both ER-α and -β [71]. Rg3 has a steroid backbone and could be a potential ligand for ER.

The mitogen-activated protein kinase (MAPK) pathway is also another regulator of eNOS (Figure 2a). Activation of MAPK signaling pathway is dependent on the extracellular stimuli and leads to cell stress response, cell proliferation, apoptosis, motility and differentiation. The MAPK family has four subgroups; the p38 group of protein kinases, c-jun N-terminal or stress-activated protein kinases (JNK/SAPK), extracellular signal-regulated kinases (ERKs) and ERK/big MAP kinase 1 (BMK1) [72]. It was shown that at 10 µg/mL, Rg3 increased the activities of c-Jun N-terminal kinase (JNK), and p38 MAPK. JNK is responsible for a number of cell functions including angiogenesis. It is responsible for a sustained phosphorylation and activation of VEGFR2 following interaction with VEGF [73] and plays a role in the phosphorylation (Ser1177) and activation of eNOS [74]. Likewise, p38 MAPK is activated by VEGFR2 and is necessary to mediate the shear stress-induced angiogenesis [75]. It also binds to and activates eNOS [76].

The other activator of eNOS is AMP-activated protein kinase (AMPK) (Figure 2a), which is a stress activated kinase. Cellular stresses such as hypoxia activate AMPK [77] followed by phosphorylation (Ser1177) and activation of eNOS [78]. Upstream of AMPK is calmodulin-dependent protein kinase II (CaMK-II). Following exposure of ECV 304 cells with 10 µg/mL Rg3, CaMK-II was phosphorylated and activated leading to activation of AMPK [21]. However, it is not yet clear if Rg3 has a similar mechanism at nM or higher µM concentrations.

### 4.3. Role of Mammalian Target of Rapamycin (TOR), Angiogenesis and Autophagy

mTOR plays crucial roles in cell growth and metabolism including lipid and protein synthesis, autophagy, mitochondrial metabolism and biogenesis, and angiogenesis. It is one of the conserved proteins belonging to the PI3K related kinase family sand downstream of activation of PI3K/Akt (Figure 2a) [79]. Activation of PI3K/Akt, both in a hypoxia-dependent and -independent manners, increases the expression of VEGF and regulated the expression of NO and other angiogenic factors. Hence, inhibitors of PI3K/Akt/mTOR pathway inhibit angiogenesis (reviewed in [80]). Cao et al. (2017) studied a rat model of endometriosis that received 10 mg/kg/day Rg3 for 21 days, resulting in blocking the VEGFR2-mediated PI3K/Akt/mTOR signaling pathway. This was evidenced by decreased protein expression of VEGF, phosphorylated Akt and phosphorylated mTOR and transcript expression of VEGF, Akt and mTOR [53]. In mice bearing breast tumors, subcutaneous Rg3 (5 mg/kg) alone or in combination with Endostar, a modified recombinant human endostatin, decreased the transcript expression of mTOR, PI3K, Akt [50], a pathway that not only is involved in the regulation of angiogenesis, but also modulates autophagy. This study also showed a decreased transcript expression JNK and of Beclin-1 [50]. JNK/Beclin-1 is a crucial pathway mediating autophagic cell death.

### 4.4. Signal Transducer and Activator of Transcription 3 (STAT3)

STAT3 is one of the important members of the STAT family which plays an important role in angiogenesis, being an activator for the transcription of VEGF [81]. Rg3 inhibited the hypoxia-induced phosphorylation of STAT3, ERK1/2 and JNK in esophageal and renal cell carcinoma lines [39].

### 4.5. TGF-β1

TGF-β1 is a member of TGF-β superfamily of cytokines. Downstream to the activation of TGF-β receptors, activation of Smads and Smad-interacting transcription factors play roles in cellular responses. Besides Smads, ERK is also activated as a part of non-Smad signaling of TGF-β receptors (Figure 2b).

Development of keloid, a hyper-proliferation in a healing wound, requires angiogenesis. Studies in keloid samples showed that Rg3 inhibited the expression of TGF-β1, VEGF and plasminogen activator inhibitor-1 (PAI-1). Smad7, a negative feedback regulator in the TGF-β1/Smad pathway, was increased and the expression levels of p-Smad2 and p-Smad3, which are enhanced by TGF-β1, were markedly decreased, p-ERK1/2 expression was decreased and the protein expression levels of total Smad2/3 and total ERK1/2 remained almost unchanged [82]. In hypertrophic scar fibroblasts RRg3 inhibited the transcript and protein expression of TGF-β1, protein levels of phosphorylated Smad2 and Smad3 and ERK1/2 and transcripts of VEGFR and platelet-derived growth factor and increased the protein level of Smad7 [41].

### 4.6. Aquaporin 1 (AQP1)

AQP1 is one of the members of water channel family of AQP proteins. It exists as a homotetramer, with every monomer responsible for the transport of water and the central channel between the four monomers responsible for the transport of ion and gases. The role of AQP1 in angiogenesis has already been discussed in the literature (reviewed in [11,83]). AQP1 plays key roles in the migration of cells, contributing to several steps including polarization, protrusion, cell adhesion to extracellular matrix (ECM), degradation of extracellular matrix and cell retraction (reviewed in [84]). Signaling of AQP1 in complex with other proteins such as focal adhesion kinase (FAK), β-catenin, Lin-7 and E-cadherin, facilitates the migration of cells (Figure 2c). Lin-7 is one of the proteins that accumulate at cadherin-catenin junctions [85]. The lin-7/β-catenin complex is also in interaction with AQP1 playing a role in the effects mediated by AQP1. Lin-7 is one of the scaffolding proteins, with the major role of assembling components of a functional complex of receptors, channels, signaling and adhesion molecules [86]. Moreover, at focal adhesion sites, integrins link the extracellular matrix and the actin cytoskeleton. FAK is another scaffolding protein functioning at these sites and regulating the interaction of proteins. It was shown that there is a functional cross talk between AQP1 and FAK. AQP1 regulates the expression of FAK and FAK colocalizes with AQP1 [87]. AQP1 also regulates the expression of β-catenin [87] and was also shown to be related to the expression of MMP-2 and -9 [88]. AQP1 also plays a role in regulating cell proliferation via regulating the expression of key cell cycle proteins such as cyclin D1 and E1 [89] and transport of oxygen reactive species (ROS), hydrogen peroxide (H_2_O_2_) [90], the signaling of which plays a role in proliferation, migration and angiogenesis [91]. In addition, increased mitochondrial ROS enhances necroptotic signaling [92] and AQP1, via effluxing ROS to the extracellular space, can potentially inhibit ROS-induced necroptosis thereby increasing cell survival.

AQP1 plays a fundamental role in the proliferation and migration of endothelial cells during angiogenesis; it is abundantly expressed in tumor microvessels and in endothelial cells in culture [83]. AQP1 has been identified as a promoter of angiogenesis [93], disruption of which impairs angiogenesis [94]. The promoter of *Aqp1* has a hypoxia response element, and following hypoxia, not only the transcription of VEGF but also AQP1 was increased [95]. This is in agreement with AQP1 as an anti-angiogenesis target. We have shown that blockers of the AQP1 water channel such as AqB013 [96], AqB050 [97] and bacopaside II [98] inhibit tube formation in endothelial cells. We have also shown that SRg3 stereoselectively inhibited AQP1-mediated transport of water [12]. Decreased expression of AQP1 with Rg3 treatment was also shown in a prostate cancer cell line [99]. This opens new windows for further investigations of the role of AQP1 as a target of Rg3 in inhibiting angiogenesis.

### 4.7. MicroRNAs (miRs)

One of the anti-angiogenic mechanisms suggested for Rg3 is via miR regulation of angiogenic pathways (Figure 2a). Keung et al. [19] screened human miR and found that in RRg3-treated HUVECs, nine miRs were differentially expressed. Based on microarray data, both hsa-miR-520h and hsa-miR-487b were increased >10 fold and hsa-miR-219, hsa-miR-342, hsa-miR-524-5p, and hsa-miR -197 were increased 2–7 fold. Additionally, hsa-miR-23a, hsa-miR-489, and hsa-miR-377 were down regulated (4 to 35 fold). In validation studies they showed a 3-fold increase in the transcripts of hsa-miR-520h in RRg3-treated cells and suggested EphB2 and EphB4 as target genes for hsa-miR-540h. EphB2 and EphB4 are two proteins of the Eph family, the largest RTK family, which upon activation mediate critical steps in cancer cell migration and angiogenesis. This study also showed that overexpression of hsa-miR-520h inhibited the proliferation and tube-forming capacity of HUVECs by 18 and 35%, respectively. Injection of hsa-miR-520h into the zebra-fish embryos showed that hsa-miR-520h significantly inhibited the neovessel formation. Knock-down of hsa-miR-520h expression significantly reduced the endogenous hsa-miR-520h level in HUVECs, their proliferation and tube-forming capacity [19]. Overall, this study showed that RRg3, potentially, via targeting hsa-miR-520h, suppressed the expression of EphB2 and EphB4 and inhibited angiogenesis.

### 4.8. CD31 and CD34

Cluster of differentiation (CD) 31 and CD34 are two of the surface molecules that have been studied as a marker of angiogenesis in many studies. These proteins are involved in angiogenesis and migration of endothelial cells. Rg3 decreased expression of CD34 in EPCs [18] and decreased expression of CD31 and CD34 in cultured patient keloid samples, by 50 and 65%, respectively [82]. Several animal studies have also demonstrated decreases in CD31 expression in tumors following treatment with Rg3 (Table 2).

## 5. Pharmacokinetic Aspects of Administering Rg3

In various in vivo models of cancer, Rg3 has been administered alone or in combination with other treatments to study the anti-angiogenic properties of this potential drug. Table 2 summarizes these studies’ doses and routes of administration and the major anti-angiogenic outcomes of the studies. These studies used doses up to 20 mg/kg and the drug was administered either p.o., i.v., intraperitoneally (i.p.) or subcutaneously (s.c.).

Depending on the structure of any drug candidate, route of drug administration might have a major role in the disposition of a drug. Among the four determinants of pharmacokinetics, absorption, distribution, metabolism and elimination, the most important determinant to consider for administration of Rg3 seems to be metabolism. From this perspective Rg3 might not be the best candidate for oral administration. It is rapidly metabolized in the gastrointestinal tract (GIT), going through partial or complete hydrolysis in the stomach and losing the sugar moieties by the GIT anaerobic microflora, leaving de-glycosylated active anti-cancer metabolites such as ginsenoside Rh2 and protopanaxadiol (PPD) [100,101,102]. Rg3 is also a substrate for cytochrome P450 members, which are abundant in the liver and GIT and also found in other organs including skin, blood, lungs and kidneys. This means that Rg3 is a potential substrate for metabolism in any of these organs [11]. Oral administration could facilitate Rg3 metabolism. However, there are controversies in the literature in terms of the concentration of Rg3 detected in the blood following oral administration. Plasma detection of Rg3 after oral administration of 10 mg/kg in Sprague-Dawley rats lasted for 12 h [103] and after 50 [104] and 100 mg/kg [105] was not detectable. The absolute bioavailability of Rg3 was calculated to be 2.63% [103]. In addition, Rg3 has a relatively high lipophilicity (estimated log P 4) (PubChem) and a low water solubility at pH 7.4 (estimated log S −4.04) (ChemAxon). These, together with the 8 H-bond donors and 13 oxygens in the structure of Rg3, make it a molecule with low permeability and low bioavailability. This also shows that Rg3 is a violation of Lipinski’s “rule of five” which makes it an inappropriate candidate for oral administration [106].

The i.p. administration bypasses the GIT metabolism, but the drug will still be exposed to the liver metabolizing enzymes before distribution in the body. Hence, i.v. and s.c. might result in more delayed metabolism and potentially a more durable action of Rg3 itself compared to the other routes of administration. However, even with a single i.v. administration, Rg3 metabolites, ginsenoside-Rh2 and protopanaxadiol, were almost instantly detected in the blood [100]. We already know that these molecules have anti-tumor and anti-angiogenic properties [107,108,109]. This raises the question, are the anti-angiogenic effects of Rg3 in vivo due to Rg3, its metabolites, or a combination of all? In that case, Rg3 is potentially not only a drug but also a prodrug.

Half-life of Rg3 following i.v. administration was studied in Sprague-Dawley rats. With 10 mg/kg, Rg3 showed a two-compartment pharmacokinetic model with half-lives of about 12 min and 2 h [103]. With 5 mg/kg, the half-life was reported to be about 14–18.5 min [104,105]. This shows that Rg3 has a generally short half-life in rats. Furthermore, the highest reported C_max_ in human study is about 400 ng/mL [110]. This is a very low concentration, equal to almost 5 × 10^−7^ nM. At this concentration, in vitro assays fail to show any efficacy of Rg3, and therefore it is possible to conclude that the efficacy of Rg3 is due to a combination of Rg3 and metabolites. This queries the sufficiency of the dosing schedule in many of the animal studies (Table 2). Administration of a single dose per day or even one dose per 3 days seems to be effective, but would they be as effective as administering 3–4 doses per day? 

## 6. Safety of Rg3

Regardless of the route of administration, Rg3 seems to be a safe drug. Acute toxicity testing of 800 and 1600 mg/kg of SRg3 (p.o.) to Sprague-Dawley rats and Kunming mice, respectively, showed no mortality or toxicity [111]. Repeated oral administration of 20, 60 and 180 mg/kg SRg3 to Sprague-Dawley rats for 26 weeks showed no sign or symptoms of toxicity, with a no-observed-adverse-effect level (NOAEL) of 180 mg/kg [111]. Another toxicity study with 7, 20, or 60 mg/kg SRg3 (p.o.) was performed on Beagle dogs for 26 weeks and showed that SRg3 was safe. The only adverse finding was the increased but reversible kidney weight in dogs that received 60 mg/kg SRg3. The NOAEL in this study was found to be 20 mg/kg [112], the human equivalent dose of which is approximately 11 mg/kg. In healthy humans receiving intramuscular injections of 10–60 mg/kg SRg3 as a single dose or 30 mg/kg once every two days for 15 days the drug was well tolerated with no detectable sign or symptoms of toxicity [110]. Furthermore, some clinical trials on non-small cell lung carcinoma [113,114] and advanced hepatocellular carcinoma [115] have used Rg3 as orally administered anti-angiogenic agent, up to 50 mg/day with no reported toxicity [113,114,115]. Therefore, Rg3 at these doses appears to be safe and well tolerated.

## 7. Conclusions

From the literature, Rg3 has been shown to inhibit the proliferation and survival of endothelial cells and the expression of various factors involved in angiogenesis. The key driver of this process is the interaction between VEGF and VEGFR2. As discussed in this review paper, several in vitro and in vivo studies showed that Rg3 decreased the expression of these two molecules, and it could be postulated that this is the major mechanism of anti-angiogenic effect of Rg3. In addition, several other mechanisms are suggested including decreased expression of b-FGF, TGF-β1, AQP1, JNK, Beclin-1, MMP-2, MMP-9 and Bcl-2. Rg3 also decreased the activation of various signaling pathways leading to activation of eNOS, including VEGF-induced Akt/eNOS, ER/PI3K/eNOS or AMPK/eNOS and decreased activation of PI3K/Akt/mTOR pathway, STAT3, ERK1/2 and JNK. It also decreased hsa-miR-520h-mediated expression of EphB2 and EphB4. With a few exceptions, studies describe this anti-angiogenic effect at µM range. Yet, some studies show Rg3 is effective at nM range too. This raises the question whether Rg3 has a biphasic or tri-phasic dose–response curve. In either case, higher efficacy of Rg3 in nM range is impressive, considering the low bioavailability following oral administration and high metabolism rate. It seems that administering the drug at µM doses leaves only nM concentrations in the blood, which is sufficient to exert the anti-cancer effects. Whether the metabolites of Rg3 also follow the same pattern is an unanswered question.

Considering the high rate of metabolism of Rg3, which leaves low levels of Rg3 in the blood, a dose-dependent anti-angiogenic effect at nM scale explains the observed in vivo anti-angiogenic effects, which could especially be potentiated by other metabolites of Rg3. Despite various in vivo reports supporting the anti-angiogenic action of Rg3, it should be taken into consideration that Rg3 is potentially a drug and a prodrug, which upon metabolism with active metabolites, ginsenoside Rh2 and PPD, could also contribute to the effects observed for Rg3. Therefore, the in vivo effects observed from this drug candidate could be attributed to a combination of Rg3 and its metabolites.

The final important issue is that Rg3 has two epimers with stereoselective activities, efficacies and pharmacokinetic profiles [100]. These epimers should be considered as two separate drugs; hence, the term Rg3 is vague and might not reflect the true nature and pharmacokinetic profile of the administered drug.

## Figures and Tables

**Table 1:** Controversies on the proangiogenic or anti-angiogenic effects of Rg3 on endothelial cells.

	Epimer	Concentration	Tested Cell	Effect	Ref
Anti-angiogenic	RRg3	1–1000 nM	HUVEC	↓ tube-formation ↓ chemotactic migration ↓ microvascular sprouting ↓ hemoglobin content of tumors	[15]
	Rg3	1.3 µM	HUVEC	↓ tube-forming capacity ↓ hemoglobin content of Matrigel plugs	[16]
	Rg3	60–600 nm/mL	EPC	↓ expression of VEGF and VEGFR2 ↓ proliferation, migration and tube formation	[17]
	Rg3	60, 300 ng/mL	EPC	inhibition of differentiation	[18]
	RRg3	100 nM	HUVEC	↑ miR-520h ↓ EphB2 and EphB4 ↓ proliferation and loop formation	[19]
Pro-angiogenic	Rg3	1–10 µg/mL	ECV 304	↑ expression and phosphorylation of eNOS ↑ expression of PI3K, JNK, p38 MAPK ↑ gene transcription mediated by ER and GR ↑ CaMK-II and AMPK	[21]
	SRg3	15 µM	HUVEC	↑ proliferation (50%) ↑ DNA synthesis ↑ migration ↑ loop formation ↑ activation of ERK/Akt/eNOS ↑activation of PPARγ	[20]
	RRg3	15 µM	HUVEC	↑ proliferation (10%) ↑ loop formation	
Anti-angiogenic	RRg3	65 µM	HUVEC	↓ tube formation and migration ↓ protein and transcript expression of VEGF, b-FGF, MMP-2, MMP-9	[22]
	Rg3	180 µg/mL	HUVEC	↓ proliferation ↓ expression of VEGF and Bcl-2 S-phase cell cycle arrest	[23]

**Table 2:** Antiangiogenic properties of Rg3 studied in different cancer models.

Cancer	Animal Model	Rg3, Dose and Route of Administration	Other Drugs in Study	Results	Ref
**Breast**	BALB/c mouse	10 mg/kg/day, p.o.	Low dose capecitabine, 200 mg/kg/day, p.o.	↓ MVD ^a^ and VEGF expression (especially in the combination group)	[49]
	Nude mouse	5 mg/kg q.a.d., s.c.	Recombinant human endostatin, 10 mg/kg, q.a.d.	↓ VEGF-A, -B, -C (especially in the combination group), proteins involved in autophagy pathway, mTOR, PI3K, Akt, JNK and Beclin-1	[50]
**Ovary**	Nude mouse	i.p.	Cyclophosphamide	↓ MVD and VEGF expression (combination)	[51]
	Nude mouse	0.3, 1 and 3 mg/kg/d for 20 days, i.p.		↓ number of vessels oriented toward the tumor mass	[52]
**Uterus**	Rats	5 or 10 mg/kg/d for 21 days	Gestrinone	Rg3 (10 mg/kg/d) + gestrinone significantly decreased the expression of VEGF, VEGFR2, p-Akt and p-mTOR, suggesting Rg3 blocks the effect of VEGFR2 via PI3K/Akt/mTOR signaling pathway	[53]
**Colorectal cancer**	Nude mouse	25 mg/kg/d for 12 days, gastric perfusion		Inhibited the expression of angiogenesis-related genes, MVD and decreased neo-vessel formation	[54]
	Nude mouse	10 mg/kg/d for 30 days, p.o.	Radiotherapy twice weekly (2 Gy) for 2 weeks	↑ effects of radiation on the expression of CD31	[55]
**Thyroid**	Nude mouse	10 mg/kg/d, intragastric		↓ CD31 in the tumors	[40]
**Lung**	Mouse	20 mg/kg/day for 18 days, (gastric perfusion)	Gemcitabine, 10 mg/kg, i.p. every 3rd day	↓ VEGF expression, MVD and signals of blood flow and peak systolic velocity of the tumor	[56]
	Mouse	600 µg/kg/day (p.o.) for 23 days		↓ arterial and capillary density, decreased number of CD34+/VEGFR2+ EPCs	[17]
	Wistar rats	1 mg/kg		↓ tumor volume and MVD	[57]
**Melanoma**	C57BL/6 mouse	1.5 mg/kg every other day for 20 days (i.v.)		↓ MVD	[58]
	C57BL/6 mouse	0.3, 1.0 or 3.0 mg/kg Rg3 (i.p.) for 10 days	5-Fluorouracil, 20 mg/kg	↓ vessel numbers, MVD and VEGF and proliferating cell nuclear antigen (PCNA)	[59]
**Liver**	A rabbit model of liver VX2 carcinoma	6 mg/kg (i.v.)	TAE ^b^	↓ CD31 and VEGF and ↑ Bcl-2 and caspase-3	[38]
	Buffalo rat	1 mg/kg (i.p.)	TAE ^b^	↓ MVD, CD31 expression, VEGF overexpression, and VEGFR2 expression and phosphorylation	[60]
	C57BL/6 mouse	10 mg/kg for 10 days		↓ MVD	[61]
**Glioma**	Rat	10 mg/kg/d for 8 days (p.o.)	LDT ^c^ 5 mg/kg/d for 8 days MDT ^d^ 30 mg/kg/d for 3 days	↑ rCBV ^e^; Untreated: 90% Rg3: 65% MDT: 64% LDT: 51% LDT + Rg3: 15%. ↓ MVD	[23]

^a^ MVD: microvessel density. ^b^ TAE: transcatheter arterial embolization. ^c^ LDT: low-dose temozolomide. ^d^ MDT: maximum-tolerated dose temozolomide. ^e^ rCBV: relative cerebral blood volume.
